# Epidemic characteristics and related risk factors of occupational exposure for pediatric health care workers in Chinese public hospitals: a cross-sectional study

**DOI:** 10.1186/s12889-019-7862-2

**Published:** 2019-11-05

**Authors:** Yuanshuo Ma, Xin Ni, Yu Shi, Chunmei Yan, Lei Shi, Zhe Li, Xiangxu Gao, Dinan Wang, Xi Yang, Lihua Fan, Yongchen Wang

**Affiliations:** 1 School of Health Management, Harbin Medical University, No.157 Baojian Road Nangang District, Harbin, 150081 China; 2 Harbin Medical University, No.246 Xuefu Road Nangang District, Harbin, 150001 China; 3 Medical Dispute Office, Beijing Traditional Chinese Medicine Hospital, Capital Medical University, Beijing, 100010 China; 4 Medical Dispute Office, Beijing Friendship Hospital, Capital Medical University, Beijing, 100050 China; 5 Department of General Practice, the Second Affiliated Hospital of Harbin Medical University, Harbin, 150001 China

**Keywords:** Occupational exposure, Pediatric health care workers, Risk factors, Epidemic characteristics

## Abstract

**Background:**

Health care workers have a high risk of occupational exposure. However, the risk of occupational exposure for pediatric health care workers has not been acknowledged in previous studies. The purpose of this study was to investigate the occupational exposure rate of pediatric health care workers in Chinese public hospitals, to explore risk factors for occupational exposure, and to put forward corresponding countermeasures to reduce occupational exposure of pediatric health care workers and protect their physical and mental health.

**Methods:**

A cross-sectional study was conducted with pediatric health care workers in 43 hospitals in 15 provinces in eastern, central, and western China between July and October 2018. With this sample, we computed the descriptive statistics of the demographic characteristics, calculated the frequency of various types of occupational exposure, and tested risk factors for occupational exposure using a chi-squared test and binary logistic regression analysis.

**Results:**

Most respondents were nursing staff (61.1%) and workers with a low-ranking professional title (50.5%). The most common style of occupational exposure in our sample was a hazard in the work environment (62.6%). Notably, physicians were less likely to experience occupational exposure than nurses (OR = 0.320, 95% CI = 0.241, 0.426). Meanwhile, pediatric health care workers who interpreted the doctor-patient relationship as harmonious (OR = 0.304, 95% CI = 0.152, 0.607) were less likely to suffer occupational exposure.

**Conclusion:**

Pediatric health care workers in Chinese public hospitals have a high occupational exposure risk and the risk factors are complex and diverse. The state, society, hospitals should acknowledge this issue and develop strategies to protect the physical and mental health of pediatric health care workers.

## Background

Occupational exposure can be defined as the presence of a substance or risk factor in the work environment external to the worker, they include physical factors, chemicals, biological agents, physical stress and psychosocial stressors [1]. In health facilities, health care workers have a higher risk of occupational exposure in their daily work [2]; their health is seriously threatened, which is a topic that has been of global concern [3]. According to the World Health Organization, 40% of hepatitis B and C viral infections and 2.5% of human immunodeficiency virus (HIV) infections originate from occupational exposure, such that medical personnel are 2–19 times more likely to acquire infectious diseases due to occupational exposure than other social groups [4, 5]. In a survey of 69 surgeons in a British hospital, Au found that the incidences of needlestick injuries among senior and junior surgeons were 29.1 and 6.59%, respectively, over 2 years [6]. A survey conducted by Frijstein between 2003 and 2010 reports that 1601 people experienced occupational exposure during this period [7]. During the 2013–2016 Ebola virus outbreak, over 890 health care workers were infected with a fatality rate of 57% [8]. Ayalu et al. found that health care workers in Ethiopia had a relatively high risk of needlestick injuries (30.5%) and sharp injuries (25.7%) [9]. Meanwhile, a survey at a university teaching hospital in mainland China uncovered that 64% of the hospital’s health care workers had experienced needle and sharp injuries [10]. Occupational exposure negatively impacts the mental health of health care workers [11], and the fear, despair, and other disabling emotions it yields in them can worsen their quality of work, potentially cultivating more negative emotions among colleagues and patients. Furthermore, hospital infections caused by occupational exposure will result in unnecessary waste in terms of the economy and resources [12, 13], which will further result in immeasurable health and social problems [3, 14–16].

It is clear that most countries recognize the dangers of occupational exposure for health care workers and establish corresponding laws and regulations to regulate and control its occurrence. In 2000, the National Institute of Occupational Safety and Health introduced “The Needlestick Safety and Prevention Act” to reduce the incidence of exposure to bodily fluids and blood among health care workers [17]. Meanwhile, the European Union, the United States, and Japan have also established relevant laws and regulations to enhance awareness of occupational safety among health care workers and to prevent and reduce the occurrence of occupational exposure [18, 19]. Moreover, to reduce the occurrence of occupational exposure incidents, China has also put forth laws and regulations, such as “Guideline on Occupational Protection from HIV Exposure for Health caer Workers” [20].

Compared with developed countries, there are some gap between Chinese health care workers in terms of working conditions, environment and facilities, and compliance with protective guidelines. Liu Xiaona et al. conducted a survey of health care workers in Beijing and found that only 46.8% of respondents had a strong understanding of occupational exposure guidelines, with only 43.6% of respondents feeling as if they had received relevant training [21]. Similarly, Zhang Min et al. surveyed health care workers in a general hospital in China and found that less than half (47%) wore gloves while performing surgery on patients, and, moreover, that the respondents demonstrated a poor understanding of source disease transmission and general preventive measures [22]. Meanwhile, Lin Chunqing and others found that participant compliance with postexposure prophylaxis guidelines was poor and identified barriers to improve compliance [23]. Today, due to social factors such as the current medical environment and doctor-patient relationship, Chinese health care workers are under great pressure [24] and face the risk of experiencing serious injuries resulting from occupational exposure. Previous research on occupational exposure mostly focuses on the impact of specific substances or the behavior of health care workers [25, 26]. Few studies examine the current elements of occupational exposure and risk factors for health care workers, and none explore occupational exposure in relation to pediatric health care workers [27–29]. Compared with health care workers in other departments, pediatric health care workers have a different working environment and clients, and are a relatively unique group in the medical team [30]. Therefore, it is necessary to investigate the occupational exposure of pediatric health care workers.

The purpose of this study was to investigate pediatric health care workers occupational exposure in secondary and tertiary hospitals in various regions of China to elucidate the characteristics of occupational exposure in Pediatrics, explore related risk factors, and suggest corresponding countermeasures.

## Method

Between July 10 and October 10, 2018, a cross-sectional study was conducted among pediatric health care workers from 43 hospitals in eastern China (Beijing, Shandong, Zhejiang, and Guangdong), central China (Heilongjiang, Henan, Hubei, and Shanxi), and western China (Inner Mongolia, Guangxi, Chongqing, Guizhou, Gansu, Qinghai, and Xinjiang) by convenience sampling. An anonymous survey was conducted through online questionnaires. We sent Web links to online questionnaires to participants via their mobile phones which respondents completed in their spare time. This type of survey method not only enabled us to monitor data collection in real time but also to effectively manage the data. In previous studies, researchers have used this survey method to complete relevant research [31]. This study obtained permission from relevant departments, hospital managers, and participants and the survey was completed with the help of Quality, Safety, Health, and Environmental Protection (QSHE) and the Management Specialty Committee Hospital Occupational Health Management Group of the China Research Hospital Association. Six tertiary hospitals and four county hospitals in Harbin, Heilongjiang Province, were selected as pre-survey units. A total of 130 questionnaires were distributed and 112 valid questionnaires were collected (these data were excluded from the formal survey). A total of 2316 pediatric health care workers participated in the formal survey and 1932 returned valid questionnaires, yielding an effective response rate of 83.42% (incomplete questionnaires, those with obvious errors, and those with a completion time < 5 min were marked invalid). The inclusion criteria of this study were as follows: (1) physician, nurses, and medical technicians working in the hospital; (2) more than 1 year of experience; and (3) voluntary participation. The exclusion criteria were as follows: (1) pediatric health care workers who were unwilling to participate in the investigation and (2) refresher pediatric health care workers and interns.

The survey was divided into two parts: the first part included the level of medical institution, type of staff, work department, working years, professional title, education level, satisfaction with the professional environment, evaluation of the relationship between doctors and patients in China, degree of satisfaction with work, and other basic information. The second part used the Nursing Career Risk Assessment Questionnaire to measure the occupational risk of pediatric health care workers in public hospitals in China. The questionnaire was based on Li Hong’s review [32] of relevant literature and related documents of the International Labour Organization Occupational Safety and Health Information Center, and has been widely used in medical environments in China. The questionnaire used in this survey was revised several times according to pre-survey results to form the final questionnaire. The final questionnaire consisted of 28 items, including 9 accidental hazards, 4 physical hazards, 4 chemical hazards, 4 biological hazards, and 7 work environment hazards. Using a 5-point Likert scale, the Cronbach’s alpha coefficient of the questionnaire was 0.94, and the reliability was good.

## Data analysis

This study used SPSS 24.0 to conduct statistical analysis. Descriptive statistical analysis was conducted for the basic demographic characteristics of pediatric health care workers and frequencies of various types of occupational exposure. A chi-square test was used to analyze the relationship between the demographic characteristics of the respondents, their subjective feelings about their living and working conditions, and whether or not they experienced occupational exposure. Taking the significant factors as independent variables and the experience of occupational exposure in the past year as the dependent variable, a binary logistic regression analysis was conducted to identify risk factors of occupational exposure for pediatric health care workers. A level of significance of *P* < 0.05 was chosen.

## Results

In terms of demographic characteristics, most participants were nurses (61.1%) or physicians (36.6%). The majority of interviewees had worked for 1–5 years (30.6%), had a primary professional title (50.5%), and had earned a bachelor’s degree (59.9%). (Table 1).

**Table 1:** Demographic characteristics of the respondents (*n* = 1932)

Characteristics	N	Percent (%)
Hospital level		
Tertiary	1803	93.3
Secondary	129	6.7
Profession		
Nurse	1180	61.1
Medical Technician	44	2.3
Physician	708	36.6
Work Experience(years)		
1–5	701	36.3
6–10	534	27.6
11–15	285	14.8
16–20	155	8.0
> 20	257	13.3
Professional title		
Primary	975	50.5
Intermediate	591	30.6
Senior	245	12.7
No title	121	6.3
Education level		
<Bachelor	391	20.2
Bachelor	1157	59.9
≥ Master	384	19.9
Marital status		
Married	508	26.3
Single	1381	71.5
Other	43	2.2

The results showed that the occupational exposure rate of pediatric health care workers in Chinese public hospitals was very high (93.0%) in the past year. In total, 62.6% (1209 respondents) believed that they were injured by the working environment, 31.0% had experienced physical hazards, 30.3% biological hazards, and 13.8% accident hazards. (Table 2).

**Table 2:** Incidence of occupational exposure in the past year (*n* = 1932)

Variables	N	Percent (%)
Accidental hazards		
exposure	267	13.8
unexposed	1665	86.2
Physical hazards		
exposure	598	31.0
unexposed	1334	69.0
Chemical hazards		
exposure	466	24.1
unexposed	1466	75.9
Biological hazards		
exposure	585	30.3
unexposed	1347	69.7
Physical and mental hazards		
exposure	1209	62.6
unexposed	723	37.4
Total		
exposure	1797	93.0
unexposed	135	7.0

Table 3 shows the correlation between the demographic characteristics of the respondents, subjective assessment of the environment, and whether or not occupational exposure had occurred. The results of the study make clear that the categories of pediatric health care workers in public hospitals in China (χ^2^ = 19.892, *p* = 0.001), working years (χ^2^ = 39.479, *p* < 0.001), professional title (χ^2^ = 43.725, *p* < 0.001), education level (χ^2^ = 12.024, *p* = 0.002), satisfaction with working environment (χ^2^ = 49.995, *p* < 0.001), awareness of self-protection (χ^2^ = 16.128, *p* < 0.001), and evaluation of the current doctor-patient relationship (χ^2^ = 25.342, *p* < 0.001) were significantly associated with occupational exposure. (Table 3).

**Table 3:** Chi-square test for occupational exposure-related risk factors (*n* = 1932)

Variables	occupational exposure		χ^2^	P
	Yes	No		
Profession				
Nurse	755	425	19.892	<0.001
Medical Technician	21	23		
Physician	385	323		
Work Experience(years)				
1–5	358	343	39.479	<0.001
6–10	337	197		
11–15	190	95		
16–20	101	54		
> 20	175	82		
Professional title				
Primary	537	438	43.725	<0.001
Intermediate	407	184		
Senior	162	83		
unrated	55	66		
Education level				
<Bachelor	206	185	12.656	0.002
Bachelor	727	430		
≥ Master	228	156		
Satisfaction with the working environment				
satisfied	446	422	49.995	<0.001
general	525	260		
unsatisfied	190	89		
Self-protection awareness				
careful	939	674	16.128	<0.001
general	203	93		
careless	19	4		
Evaluation of the current doctor-patient relationship				
tension	979	604	25.342	<0.001
general	169	134		
harmonious	12	13		

Through binary logistic regression analysis, we found that physicians (OR = 0.320, 95% CI = 0.241, 0.426) and medical technicians (OR = 0.331, 95% CI = 0.170, 0.645) were less likely than nurses to experience occupational exposure. Meanwhile, the risk of occupational exposure for pediatric health care workers with intermediate (OR = 1.726, 95% CI = 1.082, 2.753) and senior (OR = 2.139, 95% CI = 1.171, 3.905) professional titles was much higher than that of unrated pediatricians. The risk of occupational exposure for pediatric health care workers who were generally satisfied (OR = 1.885, 95% CI = 1.521, 2.336) or unsatisfied (OR = 1.766, 95% CI = 1.287, 2.423) with their occupational environment was 1.766 times higher than that of pediatric health care workers who were satisfied with their occupational environment. (Table 4).

**Table 4:** Binary logistic regression of risk factors associated with occupational exposure (*n* = 1932)

Variables	B	OR	95%CI	P
Profession				
Nurse	Ref	1		
Medical Technician	−1.106	0.331	(0.170,0.645)	0.001
Physician	−1.139	0.320	(0.241,0.426)	<0.001
Work Experience(years)				
1–5	Ref	1		
6–10	0.188	1.206	(0.926,1.571)	0.165
11–15	0.215	1.240	(0.875,1.759)	0.227
16–20	−0.040	0.961	(0.600,1.537)	0.867
> 20	0.152	1.164	(0.728,1.862)	0.527
Professional title				
Primary	Ref	1		
Intermediate	0.054	1.055	(0.702,1.585)	0.797
Senior	0.546	1.726	(1.082,2.753)	0.022
unrated	0.760	2.139	(1.171,3.905)	0.013
Education level				
<Bachelor	Ref	1		
Bachelor	0.503	1.654	(1.271,2.151)	<0.001
≥ Master	0.965	2.624	(1.762,3.907)	<0.001
Satisfaction with the working environment				
satisfied	Ref	1		
general	0.634	1.885	(1.521,2.336)	<0.001
unsatisfied	0.569	1.766	(1.287,2.423)	<0.001
Self-protection awareness				
careful	Ref	1		
general	0.364	1.439	(1.076,1.924)	0.014
careless	1.128	3.089	(0.978, 9.759)	0.055
Evaluation of the current doctor-patient relationship				
tension	Ref	1		
general	−0.228	0.796	(0.612,1.036)	0.090
harmonious	−1.190	0.304	(0.152,0.607)	0.001

## Discussion

The results make clear that pediatric health care workers in Chinese public hospitals experience a very high rate of occupational exposure (93.0%), especially when compared to the occupational exposure rates of health care workers in the Netherlands, Serbia, and other countries [7, 33] as well as that of health care workers in China’s general hospitals (82.1, 64%) surveyed by previous studies [10, 34]. Analyzing the reasons for this, the gap between pediatric health care workers in China today is more serious than that of other health care workers [30]. The ratio of Chinese children to pediatricians is 2300:1 [30], this shortage of human resources increases the personal workload of each and every pediatric health care worker, causing high-intensity diagnosis and treatment work, and has put tremendous work pressure on pediatric health care workers. Together, high-intensity workloads and tremendous work stress have created high levels of occupational exposure for these workers. In addition, this study assessed occupational environmental hazard of pediatric health care workers, which may help explain the high incidence of occupational exposure in this study compared to previous research.

The results of this study showed that pediatric health care workers experienced accidental hazards, physical hazards, chemical hazards, biological hazards, and physical and mental hazards in the working environments at rates of 13.8, 31, 24.1, 30.3, and 62.6%, respectively. Among the five types of occupational exposure, pediatric health care workers suffered the most physical and mental injuries in the working environment. Analyzing the reasons, the nature of pediatric work is different from that of adult departments. Pediatric health care workers are mostly faced with children who cannot fully express their situation. Most patients are the only children in their families, their parents often care so deeply for them that, if the treatment is expected to be inconsistent with what they think, it may be violent to the health care workers [35]. This causes a lot of psychological stress, which affects the physical and mental health of health care workers. This is also consistent with the risk factors of occupational exposure found in this study. The results of this study showed that the risk of occupational exposure is higher among pediatric health care workers in a tense doctor-patient relationship in China. Today, doctor-patient relationships in China are rather tense, and the incidence of medical disputes and hospital violence is higher than that in developed countries such as those in Europe and North America [30, 36–38]. According to incomplete statistics, the media reported a total of 7 incidents of serious violence in pediatric or children’s hospitals between January 2015 and July 2016; this total is second only to the number of incidents of violence in emergency medicine in general hospitals in China [30]. Tense doctor-patient relationships have a tremendously negative impact on the work of health care workers. Relevant research shows that to adapt to and deal with tense doctor-patient relationships, pediatric health care workers must concentrate on this matter,this continuous adaptation process will greatly increase their stress, and lead to low morale and job satisfaction [39]. When pediatric health care workers perform diagnosis and treatment in a state of high pressure and low satisfaction, their attention will inevitably be dispersed, making occupational exposure more likely. To reduce the risk of physical and mental harm to pediatric health care workers, the government should devote time, resources, and energy to eradicating tension and amplifying trust in the doctor-patient relationship, enhancing workers’ senses of security, and providing a good environment for pediatric health care workers. Moreover, individual pediatric health care workers should continue to improve their doctor-patient communication skills to prevent occupational exposure and consequent damage to their physical and mental health. The hospital should also pay active attention to changes in the psychological situation of medical personnel after exposure and provide timely psychological support and intervention to protect the physical and mental health of medical personnel.

This study also found that physicians and medical technicians had a lower risk of occupational exposure than nursing staff, at 0.320 times and 0.331 times the risk of nursing staff. This is consistent with the results of previous studies [40–42]. As front-line clinical staff, compared with physicians and medical technicians, nurses have the most contact with patients and family members; more opportunities to come in contact with sharp instruments, bodily fluids, and blood; and more onerous tasks, and no time to handle it better [43]. Irregular lifestyles, heavy workload, severe psychological stress, and lack of understanding and respect from family members of children often make nurses anxious, tired, and unable to concentrate [44], making them more likely to experience occupational exposure. Pediatric health care workers should pay attention to strengthening personal protection, diagnosing and treating patients strictly in line with standard medical practices, implementing standard preventive measures, and continuously improving their own knowledge and awareness of how to best protect themselves. The hospital should rationally allocate existing human resources, reduce the work pressure of pediatric health care workers, and provide adequate protective equipment to medical personnel to reduce the risk of occupational exposure for pediatric health care workers. This study also found that years of work were not related to occupational exposure, which is consistent with the study by Ani et al. [40] but differs from the results of Albertoni [45] and others. Most people believe that occupational exposure of health care workers is related to their work experience.

One interesting finding was that workers with a professional title were more likely to experience occupational exposure than non-professional workers, and those with higher education were more likely to experience occupational exposure than those with less education. This is not consistent with the findings of Afia Zafar [46], but is consistent with those of Yoneo and Au [6, 47]. Yoneo believes that health care workers with professional title is more likely to diagnose and treat relatively complex conditions and perform more operations, so they are more likely to have occupational exposure. As far as pediatric paramedics in public hospitals in China, most childhood diseases are urgent and change rapidly. As a result, these pediatric health care workers have more demands in terms of treatment and nursing. They have rich experience and more knowledge and highly educated health care workers are more likely to take on more tasks during the diagnosis and treatment process and to meet more patients. The accordingly intense workload and working hours these workers endure increase their risk of occupational exposure. The state should fundamentally resolve the shortage of pediatric health care workers in Chinese hospitals and optimize the training system of pediatric health care workers, gradually improve the technical level of pediatric health care workers, and build a sufficient number of skilled pediatric health care workers, so as to alleviate the personal work pressure of pediatric health care workers and reduce the risk of occupational exposure.

The satisfaction of pediatric health care workers with their occupational environment in Chinese hospitals is also an important risk factor for occupational exposure. Pediatric health care workers who are satisfied with their occupational environment have a lower risk of occupational exposure than those who are not satisfied with their occupational environment. This is consistent with the results of Mohammad et al. [48] The occupational environment of health care workers has a directly or indirectly influence on their physical and mental health [49–51]. and thereby affects their attitude and quality of work. Crowded and noisy diagnostic environments can cause bad moods and irritability, which leads to inadequate concentration of energy in daily diagnostic and therapeutic activities, work errors, and the risk of occupational exposure. In turn, occupational exposure will have a negative impact on the work, life, and physical and mental health of health care workers, increase their dissatisfaction with the occupational environment, and form a vicious circle. In response, hospitals and governments should increase their investments in manpower as well as material and financial resources to improve the occupational environment of health care workers in children’s hospitals and create a relaxed and harmonious working atmosphere, ultimately amplifying the satisfaction of pediatric health care workers with their occupational environments and reducing the occurrence of occupational exposure for pediatric health care workers.

This study also showed that pediatric health care workers with strong self-protection awareness had a lower risk of occupational exposure. This is consistent with the findings of Jankovic [52]. Health care workers with strong self-protection awareness will pay more attention to their diagnosis and treatment operations in daily medical activities, strictly abide by the routine of diagnosis and treatment, and have higher vigilance for occupational exposure, so the risk of occupational exposure is lower. Research shows that occupational safety education for health care workers is an effective way to reduce occupational exposure [53, 54]. Accordingly, Hospitals should strengthen training in occupational protection for medical and health personnel [55] and medical schools should optimize clinical occupational protection education [33] to ensure that health care workers gain systematic and comprehensive knowledge of occupational exposure to improve their sense of occupational protection, standardize their diagnosis and treatment operations, and reduce the occurrence of occupational exposure.

This article has several limitations. First, the survey collected data on occupational exposure over the past year, which may have caused reminiscent bias. Second, because of the cross-sectional study design, the causal relationship may not be determined. Third, this study adopted a convenient sampling method, which may have led to large deviations in results and insufficient sample representation.

## Conclusions

The incidence of occupational exposure of pediatric health care workers in Chinese hospitals is high and the risk factors are complex and diverse. Most pediatric health care workers suffer from physical and mental injuries resulting from the conditions in which they work and live. Their work nature, professional title, education level, satisfaction with the working environment, evaluation of doctor-patient relationship, and self-protection awareness are all risk factors for occupational exposure. The government, hospitals, medical colleges, and medical personnel must actively pay attention to this problem and take effective measures to reduce the incidence of occupational exposure for pediatric health care workers to protect their physical and mental health.

## Data Availability

The datasets and/or analyses from the current study will be available from the corresponding authors upon reasonable request.

## Abbreviations

HIV: Human Immunodeficiency Virus
